# *In situ* formation of PLA-grafted alkoxysilanes for toughening a biodegradable PLA stereocomplex thin film

**DOI:** 10.1039/c9ra03299a

**Published:** 2019-07-15

**Authors:** Jieun Jeong, Muhammad Ayyoob, Ji-Heung Kim, Sung Woo Nam, Young Jun Kim

**Affiliations:** Department of Chemical Engineering, Sungkyunkwan University Natural Science Campus, 2066, Seobu-ro, Jangan-gu Suwon 16419 Republic of Korea youngkim@skku.edu

## Abstract

Poly(lactide) (PLA) has received tremendous attention recently from researchers and industrialists due to its ability to solve environmental problems related to plastic pollution. However, PLA's brittleness, poor thermal stability, low elongation at break, and poor melt processing prevent its use in a broader spectrum of applications. Herein, we produced a very tough and thermally more stable PLA stereocomplex by simply mixing PLA with organoalkoxysilane. The stereocomplex PLA/silane (sc-PLA–silane) composite was prepared by simple mixing of three types of organoalkoxysilanes in sc-PLA followed by *in situ* formation of a silane-based rubbery core with a cross-linked PLA shell. Mechanical and thermal properties were improved by stereocomplexation of PLA with a small amount (1–5 wt%) of PLA-grafted silanes. The addition of organoalkoxysilane with different functional groups resulted in a plasticizer of rubbery silica-PLA core–shell gel through *in situ* condensation and grafting of long PLA chains at the interface between the stereocomplex and silane particles. The results revealed that the toughness of sc-PLA was improved dramatically with only a small addition (only 2.5%) of 3-(triethoxysilyl)propyl isocyanate (ICPTES). The morphology and mechanical and thermal properties of the toughened stereocomplex films were characterized. The results revealed that elongation at break was increased from 16% to 120%, while other mechanical properties such as tensile strength and modulus were retained. Surface analysis confirmed that this toughness was achieved by formation of a silica-PLA core–shell gel. The mechanical properties of PLA were improved without any significant reduction in modulus and tensile strength using this simple methodology.

## Abstract 2

Current work provides a synergistic approach to prepare super tough PLA without any significant loss of its excellent intrinsic mechanical properties.

## Graphical abstract

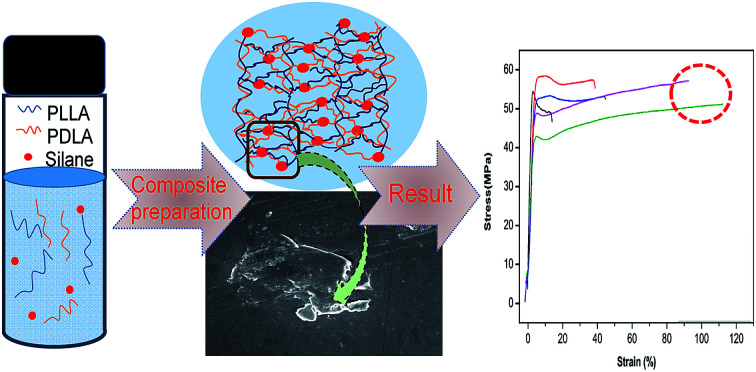

## 1. Introduction

Petroleum-based thermoplastic polymers are ubiquitous in today's world. However, the environmental issues due to plastic waste caused by petroleum-based non-biodegradable polymers have prompted researchers to find environmentally friendly sustainable alternatives. The inherent environmental sustainability, biodegradability, and renewable raw material of poly(lactide) (PLA) has increased the use of this thermoplastic polymer in the 80 years since it was first discovered by Carothers.^1^

In the last five decades, methods have been developed to improve the mechanical properties and toughness of PLA. PLA's biodegradability once considered a drawback, is now a salient feature as bioplastics are considered alternatives to petroleum-plastics.^2–4^ PLA is an environmentally friendly, biocompatible, and biodegradable thermoplastic polymer. Thus, PLA is attracting attention and has the potential to reduce environmental pollution.^5,6^ Nevertheless, the brittleness of PLA is a main hindrance, along with thermal limitations.^7–10^

PLA is an optically isomeric polymer, having d(+) and l(−) stereoisomers. The polymers made from l and d forms considerably differ in thermal properties. Thermal properties, such as thermal stability and glass, crystallization, and melt transitions, can be modified using stereoselective polymerization and by blending d and l in different proportions.^11^ The mechanical properties of PLA can also be improved by stereocomplexation.^12–14^ Stereocomplexed PLA is a suitable polymeric material for certain applications such as drug carriers, wound dressing, biomedical implants, and scaffolds for tissue engineering.^15,16^

PLLA and PDLA have optical activity due to their chiral structures. The melting temperatures (*T*_m_) of PLLA and PDLA do not exceed 180 °C. However, *T*_m_ drops quickly, and the crystallization capacity decreases sharply with a decrease in optical purity.^12,17^ The low thermal stability limits thermal processes like injection molding and extrusion.^18^ However, the melting temperature can be increased from 180 °C to about 230 °C through stereocomplexation of PLLA and PDLA.^18,19^ PLA is an inherently brittle polymer, which has 4–7% elongation at break; however, formation of a stereocomplex film with PDLA by solution casting has shown a 12% elongation at break.^20^ The improvement in toughness is due to the presence of a higher density of inter-crystalline connections within a mobile amorphous phase of the PLLA/PDLA stereocomplex film.^21^

Researchers have been trying to overcome these drawbacks of PLA while investigating different approaches to improve the mechanical properties and toughness; such methods include addition of plasticizers,^22–24^ PLA blending with reactive or modified toughening agents,^25–28^ block and random copolymerization,^29,30^ and addition of nanofillers.^31,32^ The toughness and ductility of PLA have been improved through these approaches. However, increase in ductility or toughness decreases tensile strength and modulus in most cases. For example, PLA/crosslinked polyurethane was used as a plasticizer to increase elongation at break by 38 times that of neat PLA, with considerable decrease in tensile strength and modulus.^22^ Toughness of PLA was also enhanced by epoxidized soybean oil, PLA plasticized with epoxidized soybean oil showed an increase in strain by 63% at the expense of a reduction in tensile strength up to 27%.^23^ PLA was plasticized using *in situ* reactive blending with a PEG-diacrylate monomer to improve its toughness in a study by Fang *et al.*, and the results revealed that tensile strength was dropped to 50 MPa from 75 MPa; however, elongation at break increased by 160%.^25^

Typically, toughening involves stress yielding and/or craze formation along with free volume changes.^33^ The addition of a rubbery phase and/or a plasticizer creates free volume and facilitates chain segmental motions. This enables chain sliding, which lowers the stress required for these processes. Therefore, addition of a rubbery phase or plasticizer often results in a decrease in glass transition temperature (*T*_g_) and tensile strength of the materials. In contrast, plasticizers and additives are mostly not miscible and incompatible with the PLA main matrix, which leads to phase separation and weak interfaces in the mix and further decrease in strength.^24^

Several studies suggested that polymer toughness can be enhanced by addition of alkoxysilane.^32,34–38^ Alkoxysilanes have long been in use as nanofunctional-material formers within the sol–gel process. In the sol–gel process, alkoxysilanes go through hydrolysis and condensation and eventually form silica gels through Si–O–Si bonds.^38–40^ Recently, a simple methodology has been introduced to improve toughness without any loss of tensile and modulus. The addition of silane to PLA can increase the toughness of PLA. Xiang Tao *et al.* introduced a super-tough PLA–silane nanohybrid system.^34^ Their results confirmed that addition of alkoxysilane to PLA improves the toughness with a considerable compromise in the tensile strength and modulus.^34^ Silanes with a functional end group that reacts with the PLA matrix can be added. These silanes can increase the degree of cross-linking in the interface region and react with the hydroxyl (–OH) end groups in the PLA. In this process, a PLA-grafted silane nano/micro-gel can be prepared with a PLA–silane interface through condensation.^34,35,41^ The polysiloxane formed through condensation and grafted with PLA has rubbery characteristics and acts as a plasticizer in the PLA matrix. The silica-PLA core–shell gel can improve the mechanical properties of PLA.^34,42^ PLA-grafted polysiloxane have very good miscibility, dispersibility, and compatibilization, which can help to improve the toughness and tensile strength. The effective dispersion helps to improve the mechanical properties throughout the PLA matrix.

In the present work, we combined the stereocomplex and PLA-grafted polysiloxane to obtain a synergistic effect of both to afford a mechanically strong and tough PLA for biomedical as well as packaging applications. The stereocomplex films of PLLA/PDLA were prepared by solution casting with addition of three different alkoxysilanes with different ratios. The prepared films were characterized by measuring the tensile strength and using dynamic mechanical analysis (DMA) to study the mechanical performance of the PLA stereocomplex. The thermal properties were characterized by differential scanning calorimetry (DSC) and thermogravimetric analysis (TGA) to analyze the effect of silane on the PLA. The morphologies of the PLA stereocomplex were studied by scanning electron microscopy (SEM) and FTIR spectroscopy.

## 2. Experimental

### Materials

d-(+)-Lactide (>99.5% by GC) was purchased from Tianjin ExceedBio Co., LTD. l-(−)-Lactide monomer, tin(ii) 2-ethylhexanoate (Sn(Oct)_2_), and 1-dodecanol were purchased from Sigma Aldrich. (3-Aminopropyl)triethoxysliane (APTES), 3-(triethoxysilyl)propyl isocyanate (ICPTES), and trimethoxymethylsilane (MTMS) were also purchased from Sigma Aldrich. Chloroform and methanol (>99.5%) were obtained from Daejung Chemical & Metal Co., Ltd. Tin(ii) 2-ethylhexanoate (Sn(Oct)_2_), 1-dodecanol, and chloroform were distilled before use.

### Synthesis of PLLA and PDLA

PLLA and PDLA was prepared using ring-opening polymerization as reported previously, briefly described here.^43^ In a typical synthesis procedure, monomer (l-(−)-lactide or d-(+)-lactide) (50.0 g, 0.347 mol), 1-dodecanol (0.077 g, 0.413 mmol), and Sn(Oct)_2_ (0.05 wt%) were charged into a torch-dried, three-neck flask equipped with a mechanical stirrer in a glove box under an argon environment. The flask was vacuumed and flushed with dry nitrogen five times and then sealed under vacuum. The flask was then immersed in a preheated oil bath at 140 °C. The reaction mixture was heated for 10 hours at 140 °C. The reaction was stopped by removing the flask from the oil bath and cooling it to room temperature. The resulting product was dissolved in dry chloroform and recovered by precipitation with chilled methanol. White solid PLA polymer was filtered and dried for 24 hours under vacuum at 80 °C and molecular weight was characterized using GPC.^43^

### Preparation of PLLA, PLLA/PDLA stereocomplex, and stereocomplex–silane blended films

All films were prepared using solution casting technique in chloroform. PLLA and PDLA (1 : 1) were dissolved in chloroform at about 1 : 10 w/v. A predetermined amount and type of silane was added to the PLA solution as reported previously.^44^ The PLA–silane mixture was stirred vigorously overnight at 400 rpm at room temperature. Overnight stirring promoted homogenous distribution of silane in the PLA matrix at room temperature. The solution was then sonicated for another 1 hour in a bath sonicator to improve the dispersion of PLA-grafted silane hybrid gel in the PLA stereocomplex matrix as reported elsewhere.^45^ The resulting solutions were transferred to the glass plate, and the solvent was allowed to evaporate while the silane-gel settled for 2 days. The films were then dried under vacuum at 60 °C overnight and for 5 min at 130 °C. Dried films were cooled to room temperature and stored in a dry environment at room temperature.

### Characterization

Molecular weights of the synthesized PLLA and PDLA were characterized using GPC to obtain the number-average molecular weight 
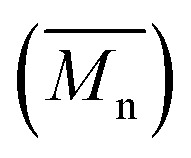
, weight-average molecular weight 
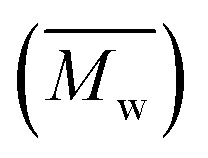
 and polymer dispersity index (PDI). GPC measurements were carried out using a Waters Alliance e2695/2414D equipped with Shodex columns of KF805, 804, and 802.5 run at 40 °C with tetrahydrofuran (THF) as an eluent at flow rate of 1.0 mL min^−1^. The universal calibration curve was constructed using polystyrene standards (Agilent EasiCal PS-1).

The mechanical properties of the PLA films were characterized using a universal testing machine (UTM) and dynamic mechanical analysis (DMA). In the tensile testing analysis, a load of 250 N from a Lloyd/LR30K at room temperature (25 ± 2 °C) was applied. All films were stored in a standard environment for 24 hours before testing and were cut into 50 mm × 10 mm × 0.1 mm rectangles. The distance between clamps was 30 mm, and a load of 250 N was employed at a speed of 5 mm min^−1^. Eight samples for each composition were tested, and the results were compiled. All collected data were averaged, and stress–strain curves were plotted. Tensile strength and modulus were calculated. Dynamic mechanical analysis was performed using a TTDMA made by Triton Corp. All films were cut into 2 mm × 20 mm pieces. During the DMA analysis, the samples were loaded and heated with a heating rate of 2 K min^−1^ from 10 °C to 170 °C. The employed strain rate was 0.1% at a frequency of 10 Hz, which was in the linear viscoelastic region (as determined by a strain sweep). For DMA analysis, all films were pre-dried for 24 hours under vacuum at 60 °C.

The thermal transitions were characterized by DSC using an AutoQ20 made by TA Instrument USA. Samples were pre-weighed and loaded in the sample pan of the instrument. Samples were heated at a rate of 10 °C min^−1^ from 25 °C to 250 °C, cooled to 25 °C, and reheated to 250 °C in a dry nitrogen atmosphere and the presented results are from 2^nd^ heating. Thermal stability was characterized using a TGA made by TA Instrument USA. The samples were loaded in the sample pan and heated at 10 °C min^−1^ to 600 °C under a nitrogen atmosphere.

Stereocomplex film morphologies were characterized using scanning electron microscopy (SEM) (JSM-7600F) to observe the morphology of surface and fractured surface at a power of 5.0 kV. All samples were coated with gold layer. Fourier transform infrared spectroscopy (FT-IR) spectra were recorded using a NicoletTM iSTM 50. Spectra were recorded using a spectral width ranging from 500 to 3500 cm^−1^ and an accumulation of 32 scans.

## 3. Results and discussion

### Polymerization and molecular weight of PLLA and PDLA

Poly(l-lactide) (PLLA) and poly(d-lactide) (PDLA) were synthesized to prepare tough stereocomplex PLA films. PLLA and PDLA were synthesized by ring-opening polymerization, and the results are presented in Table 1. The theoretical target molecular weight was in the range of 1.00 × 10^5^ to 1.50 × 10^5^, which is considered suitable for evaluating the mechanical properties of stereocomplexes. A previous report showed that good mechanical properties of PLLA and PDLA required a minimum molecular weight of about 1.00 × 10^5^ g mol^−1^.^46,47^ The molecular weights of the prepared PLLA and PDLA by GPC were 1.27 × 10^5^ and 1.31 × 10^5^ g mol^−1^ with PDI values of 2.1 and 2.2, respectively.

**Table 1:** Molecular weight information of PLLA and PDLA

Sample	*M*_n_ a (g mol^−1^)	*M*_w_ a (g mol^−1^)	PDI	*T*_m_ b (°C)	Δ*H*^b^ (J g^−1^)
PLLA	5.8 × 10^4^	1.27 × 10^5^	2.17	177.1	42.4
PDLA	5.9 × 10^4^	1.31 × 10^5^	2.22	173.2	46.5

a Determined by GPC measurement.

b Determined by DSC analysis.

### Confirmation of stereocomplex formation *via* thermal analysis

**Fig. 1:**
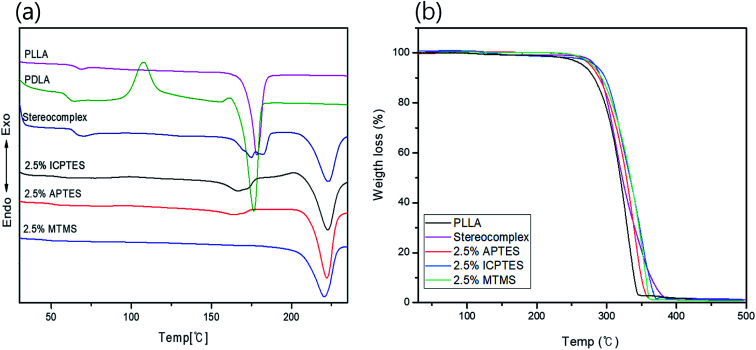
Thermal properties analysis of stereocomplex/silane blend films: (a) DSC curves; (b) TGA curves for stereocomplex/silane blend films.

Thermal transitional properties were characterized by DSC analysis of PLLA, PLA-stereocomplex and PLA/silane blends and results are presented in the Fig. 1a and Table 3. First transition was observed around 63 to 65 °C is referred as glass transition *T*_g_. *T*_g_ of all specimens was comparable and was in the range of *T*_g_ of PLA. There was no considerable change in the *T*_g_, a slight decline in *T*_g_ about 1–2 °C was noted after addition of silane 2.5% and over. A second transition was only observed in the thermograms of stereocomplex PLA at 117 °C during 2^nd^ heating is corresponding to crystallization transition (*T*_c_). The neat homo-PLLA exhibit a melt transition (*T*_m_) at 178 °C, whereas, stereocomplex melt transition (*T*_sc_) was observed at 220 °C. The increase in melting temperature is the indication of stereocomplex formation. However, two melt transitions were observed from the thermograms of stereocomplex PLA even after addition of silane except MTMS, *T*_m2_ is assigned to stereocomplex and *T*_m1_ at 178 °C was supposed due to imperfection in PLA stereocomplex formation or the PLA part that does not participate in stereocomplexation as in case of higher molecular weight, chain entanglement can have increased due to higher viscosity and resulted in less perfect stereocomplex formation.^12^ During heating, the less perfect crystals melt first at relatively lower temperature, whereas, all crystallites melted at 220 °C. No considerable changes were observed in the DSC thermograms of the PLA films with addition of 2.5% of any of the three types of silanes except MTMS, where a lower *T*_sc_ peak was disappeared perhaps due to the rubbery nature and unavailability of functional groups on MTMS silane which restricted the thinner lamellae formation. The results are shown in Table 2.

**Table 2:** Mechanical properties of stereocomplex/silane blending

Composition	Maximum tensile strength (MPa)	Young's modulus (GPa)	Strain at break (%)
PLLA	49.99 ± 3.79	20.91 ± 0.30	7.20 ± 5.2
Stereocomplex	55.68 ± 4.33	20.73 ± 0.41	16.46 ± 4.42
ICPTES 1%	57.54 ± 4.19	19.25 ± 0.44	38.71 ± 5.63
ICPTES 2.5%	49.55 ± 2.97	18.76 ± 0.86	89.18 ± 4.96
ICPTES 5%	42.39 ± 2.44	16.54 ± 0.35	92.94 ± 9.47
APTES 2.5%	55.69 ± 3.22	20.29 ± 0.88	33.08 ± 4.59
MTMS 2.5%	52.09 ± 4.10	18.85 ± 0.82	38.64 ± 6.32

**Fig. 2:**
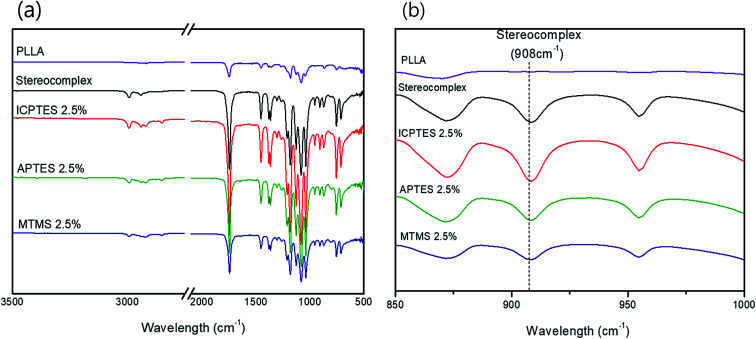
(a) FTIR spectra of PLLA, PLA stereocomplex and all three different types of 2.5% composites; (b) FTIR spectra showing characteristic absorption bands of stereocomplex.

The TGA traces in Fig. 1b show the thermal stability of the stereocomplex and PLA grafted-silane blended films. The TGA traces revealed the thermal stability of the stereocomplex and stereocomplex/silane blends was increased slightly in a comparison to homo PLLA. From TGA traces, a noticeably higher thermal stability of stereocomplex and silane blends was observed. However, it was noticed that thermal degradation of PLLA was started at 263 °C, whereas, that of stereocomplex PLA and silane/stereocomplex blends was started at a temperature over 275 °C. It is clear from Fig. 2b that the thermal stability of the PLA films was improved after stereocomplex formation and even with addition of PLA-grafted silane did not change much.^34^ This result implies the formation of stereocomplex microstructural changes within the PLA matrix. The stereocomplex barrier can prevent heat and mass transmission during decomposition in the main matrix. However, the degradation behavior of the stereocomplex and silane blending films is complex due to the intermolecular and intramolecular transesterification and racemization.^48^

### Stereocomplex confirmation through FT-IR spectroscopy

The stereocomplex structure formation and addition of PLA-grafted silanes can also be confirmed from FT-IR analysis. Fig. 2a confirms no considerable structural change in the spectra of stereocomplex/silane blends. However, absorption at 908 cm^−1^ represents the presence of stereocomplexation formation, as shown in Fig. 2b. No specific differences between stereocomplexed PLA and silane-toughened PLA were observed from the FTIR spectra because the amount of silane was very small (<5% (w/w)).

### Mechanical properties of stereocomplex/silane agent blended films

**Fig. 3:**
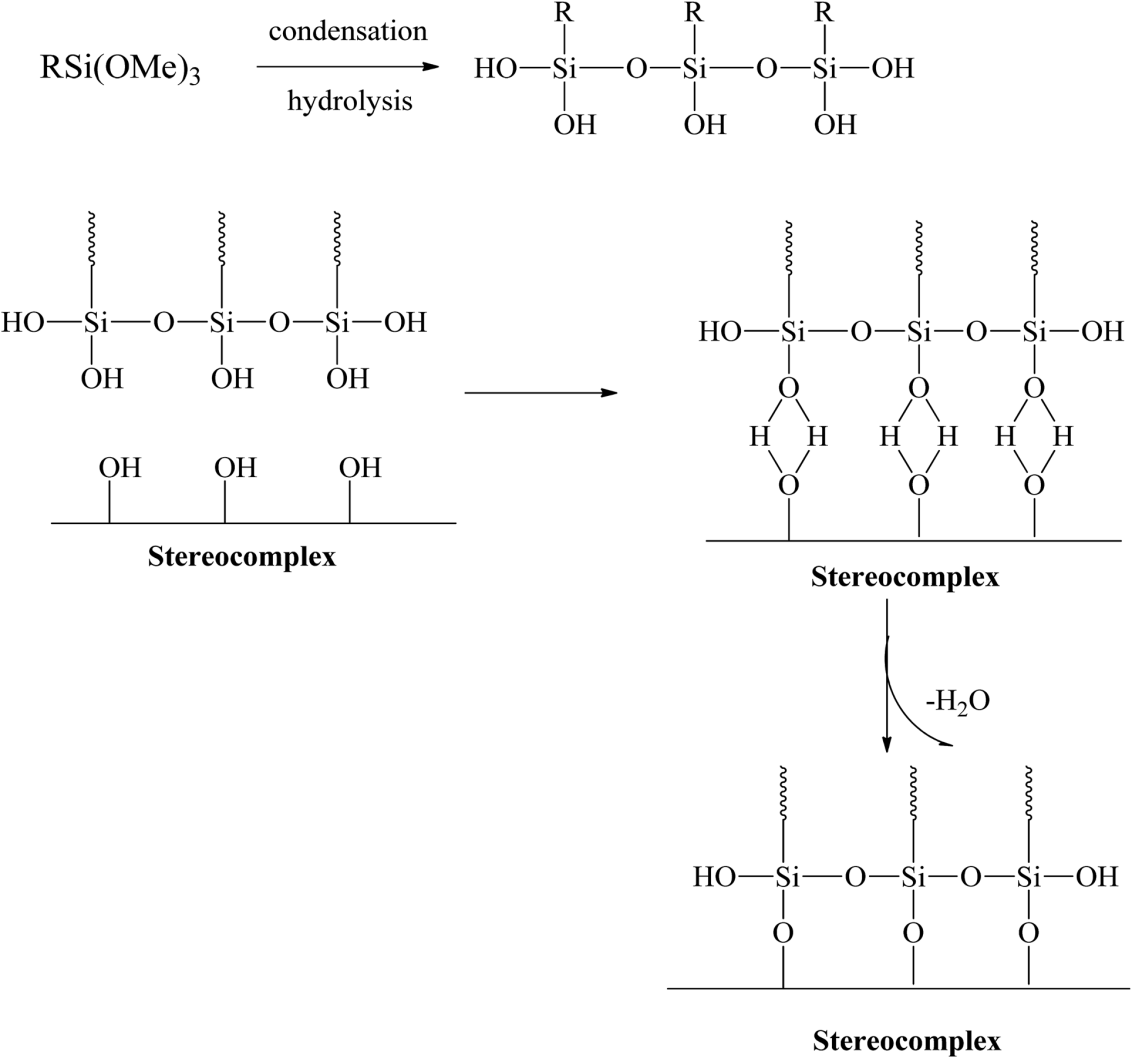
Scheme of polysiloxane formation between stereocomplex and silane agents.

Extensive efforts have been made to modify the mechanical performance of PLA using plasticizers, blends, and composites. However, the present method is a very simple and effective way to improve the ductility and toughness of PLA without any considerable loss in strength. Testing was conducted for stereocomplex films and stereocomplexes/silane agent blended films. In this study, three different types of functional silanes were blended with stereocomplexed PLA by the solvent casting method at room temperature. The proposed scheme and the resulting structure are shown in Fig. 3.

**Fig. 4:**
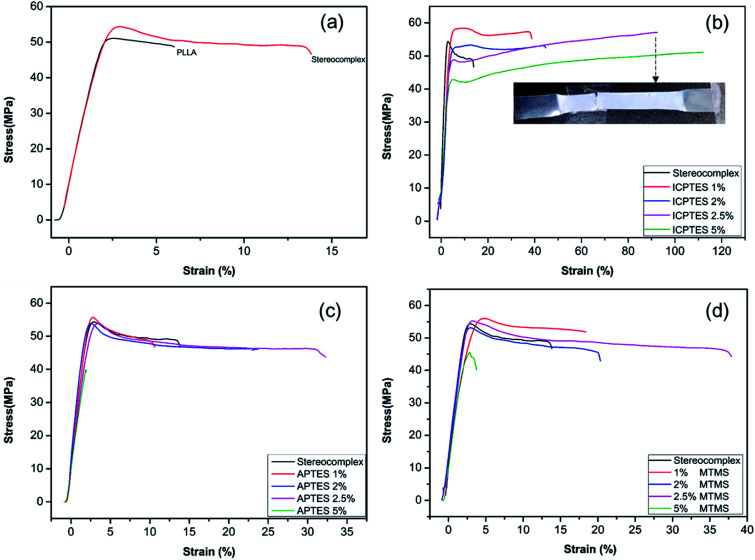
Stress–strain curves for PLLA and stereocomplex/silane blend films: (a) PLLA and stereocomplex; (b) stereocomplex/ICPTES composites; (c) stereocomplex/APTES composites; (d) stereocomplex/MTMS composites.

The films were prepared with different loading percentages of silanes, and samples tested using UTM for tensile properties and the corresponding results are presented in Fig. 4 and Table 3.

**Table 3:** Thermal properties of stereocomplex/silane blending

Composition	*T*_m_ (°C)	*T*_g_ (°C)	*T*_c_ (°C)	Δ*H* (g J^−1^)
Stereocomplex	221	65	117	44.3
ICPTES 1%	219.4	—	131	54.2
ICPTES 2.5%	220.4	—	147.6	57.3
ICPTES 5%	220.4	—	149.6	65.4
APTES 2.5%	223	—	101.5	55.9
APTES 5%	223	—	114.5	46.7
MTMS 2.5%	222	63	103.5	55.6
MTMS 5%	221	—	102	45.3

Fig. 4a shows the mechanical properties in the form of a stress–strain plot of neat PLLA and the stereocomplex. There were no significant differences between the modulus values of PLLA and the stereocomplex. However, the tensile strengths of the PLLA and PLA stereocomplex were 50 MPa and 55 MPa, respectively. Similarly, the strain was notably higher for the stereocomplex than the PLLA. The elongation at break was 5–7% and 15–17% for the PLLA and stereocomplex, respectively (Fig. 4a). These results confirmed that the mechanical and thermal properties of the stereocomplex were enhanced compared to those of PLLA. The improvements in mechanical properties were attributed to the presence of a higher density of intercrystalline connections within the mobile amorphous phase.^21,49^ Both PLA and PLA stereocomplexes exhibited brittleness and broke without necking.

Alkoxysilanes improved the elasticity and ductility of PLA, as was already reported, but the stress was considerably affected. Here, we report a comparatively higher toughness with improved tensile stress by synergistically using silanes as a rubbery gel in the stereocomplex PLA matrix. The results indicate a mechanical transition of the silane/PLA composite from brittle to ductile with remarkable improvements in the toughness with small amounts of alkoxysilane. Fig. 4b shows the stress/strain plot of stereocomplex PLA/ICPTES composites. The amount of ICPTES used to prepare the composite stereocomplex PLA was very low (from 1 to 5% (w/w)) because a larger amount of silane causes aggregation within the polymer matrix and reduces the properties.^36^ Thus, up to 5% silane is appropriate to enhance the mechanical properties. As shown in the results, the elongation at break increased by adding 1 to 5% ICPTES.

The PLA stereocomplex showed some deformation but still exhibited a brittle nature. However, addition of only 1% ICPTES in the composites increased the yield by up to 40%, with a slight increase in tensile strength to 58 MPa, which is almost 3 times that of the PLA stereocomplex. On the other hand, there was no significant loss in tensile strength or Young's modulus. The ductility and deformation increased with an increase in ICPTES in the composite. The PLA/silane composite was very tough, as 90% elongation was achieved without any considerable loss in tensile strength or modulus. This high toughness was attained at only a limited cost of 3 MPa. That is, the tensile strength was 53 MPa, compared to the 56 MPa of the stereocomplex PLA. This trade-off was acceptable because it provided high deformation at the minimum tensile strength loss. However, toughness with 5% addition of ICPTES in the stereocomplex PLA film was not good compared with the 2.5% ICTPES composites film. Although elongation at break was much higher than that of the 2.5% ICPTES composite, the stress decreased to 42 MPa from 55 MPa. As a result, the toughness of the composite was lower than that at a 2.5% addition.

Similarly, tensile results confirmed increased deformation with an increase in the amount of APTES alkoxysilane. In contrast to the ICPTES, the values of deformation were very low. However, the stress remained unchanged. Elongation at break was obtained up to a maximum of 33% with 2.5% addition of APTES in the composite. The mechanical properties deteriorated with an increase up to 5% alkoxysilane. This was likely due to aggregation of the silane within the PLA matrix to form larger particles. The aggregates underwent a scissor-like reaction in cross-linked chain. As a result, the properties decreased dramatically. Experiments showed that the strain increased without change in stress when the amount of APTES was added up to 2.5%, but it decreased dramatically when the quantity of APTES was increased beyond 2.5%, as shown in Fig. 4c.

Similar behavior was observed with the addition of MTMS type alkoxysilane in the composite (Fig. 4d). The mechanical properties of the stereocomplex composite increased with an increase in alkoxysilane addition up to 2.5%. The mechanical properties were improved due to the reaction of hydroxyl groups with the longer polymer chains of PLA of the stereocomplex and the functional groups of the silanes to form a rubbery core–shell network within the PLA stereocomplex matrix.^34,42^ This cross-linked network of silane improved the degree of cross-linking in the interface region, which improved the properties of the PLA composites.

Cross-linking to form the PLA-grafted core–shell rubbery gel and dispersion of the gel in the stereocomplex matrix are two important factors to improve the toughness of the composite. Poor dispersion of rubbery gel and aggregation of silanes negatively affect the mechanical properties. Larger particle sizes reduce the interactions between silane and PLA matrix, resulting in decrease in mechanical properties. In the current study, ultra-sonication was used to effectively disperse the gel in the PLA matrix.

Further, we confirmed that ICPTES alkoxysilane efficiently reacted with stereocomplexed PLA in comparison to the other two types. ICPTES reacted with PLA molecular chains in the stereocomplex and formed a cross-linked network of nanofibrils, as shown in the SEM images. However, APTES produces a less dense network and larger silane–PLA rubbery particles, which reduce the compatibility with larger quantities of alkoxysilane. In contrast, MTMS does not have any specific functional groups. Therefore, only silane molecules react with other silane molecules by condensation. As a result, no cross-linked network was formed; only silane particles were observed in SEM. Thus, the isocyanate functional groups of ICPTES resulted in effective toughness of the PLA stereocomplex.

**Fig. 5:**
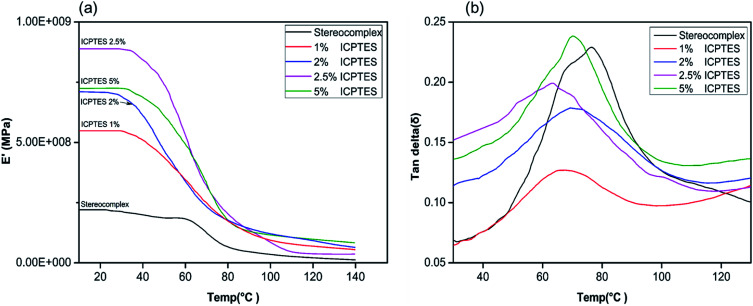
DMA thermograms of stereocomplex and ICPTES blend films: (a) storage modulus *vs.* temp. (°C); (b) tan *δ vs.* temp. (°C).

Changes in the macromolecular structure of a polymer can modify the topological properties of a polymer system, including chain entanglement and morphology. Such changes will be demonstrated in the rheological behavior of the polymer or polymer composite.^50^ Viscoelastic properties of the silane/PLA stereocomplex composite films were also studied using DMA, and the results are shown in Fig. 5. Only the top-performing silane among the three was characterized by DMA, and results showed the effect of temperature on the storage modulus of the silane/PLA stereocomplex. Stereocomplex films containing ICPTES exhibited a higher storage modulus than the lone stereocomplex. All compositions showed a higher storage modulus than that of the neat PLA stereocomplex. Specimens of all ICPTES ratios showed a single relaxation process in a temperature range of 30–120 °C, attributed to the mobility of PLA stereocomplex chains with an increase in temperature. A major drop in the storage modulus was observed at 50 to 70 °C and corresponded to the glassy region of the composite. This decrease in storage modulus was due to softening of PLA with increasing temperature. Therefore, a sharp decline was observed at 50 to 70 °C, achieving a temperature higher than the softening temperature of PLA. The PLA stereocomplex composite with 2.5% ICPTES possessed the highest storage modulus, which decreased with increasing quantities of silane. This means that the addition of a larger quantity of ICPTES had a negative effect on the storage modulus, which is attributed to the increased flexible PLA-grafted-silane rubbery gel in the composite films.

Moreover, the tan *δ* plot of all ratios of composite indicate the maximum loss at the glass transition temperature. As shown in Fig. 5, the tan *δ* intensities for 1%, 2%, and 2.5% decreased compared to that of the neat PLLA stereocomplex, whereas the peak position was the same. As the height of the intensity is directly affected by the chain mobility in the amorphous region, this loss in intensity indicated that addition of silane rubbery gel had no effect on the *T*_g_ of the composite. However, it affected the mobility of PLA chains significantly, resulting in reduction in tan *δ* intensity.^42^ Addition of 5% silane-rubbery gel in the composite resulted in a reduction in *T*_g_ of the composite. In the glassy region, ICTPES 5% composite films exhibited the highest storage modulus, while stereocomplex PAL films had the lowest storage modulus. Likewise, in Fig. 5b, the tan *δ* curve of 5% ICTPES films showed the highest peak intensity, which indicates that soft and flexible rubbery segments of silanes lowered the *T*_g_ and increased the chain mobility. As a result, the chain mobility increased due to the subtle changes in the macromolecular motions of PLA molecules in the glassy region. The glass transition temperature values of DMA and DSC were different but within the expected range.^42^

In the present work, toughness of PLA was improved significantly with the synergistic effect of intrinsically strong mechanical properties of PLA stereocomplex and plasticity of PLA-grafted-silane core–shell type rubbery particles. Contrary to previously reported results, we present an elongation up to 90% without any considerable loss in tensile strength as shown in Fig. 5b with addition of 2.5% ICPTES. The yield strength was decreased about 4 MPa only, from 53 to 49 MPa, whereas, the maximum stress at break was 55 MPa. On the other hand, at the cost of 20 MPa reduction in tensile strength, from 60 to 40 MPA, the elongation at break was increased up to 130%.^34^ Similar results were reported by Zibiao Li *et al.* where tensile strength was dropped 20 MPa, from 50 to 30 MPa.^42^ We strongly believe that this high tensile strength is the result of co-crystallization of PLA chains which are grafted on the silane rubbery particles with in the stereocomplexation of PLLA and PDLA.

### Morphology analysis by SEM

**Fig. 6:**
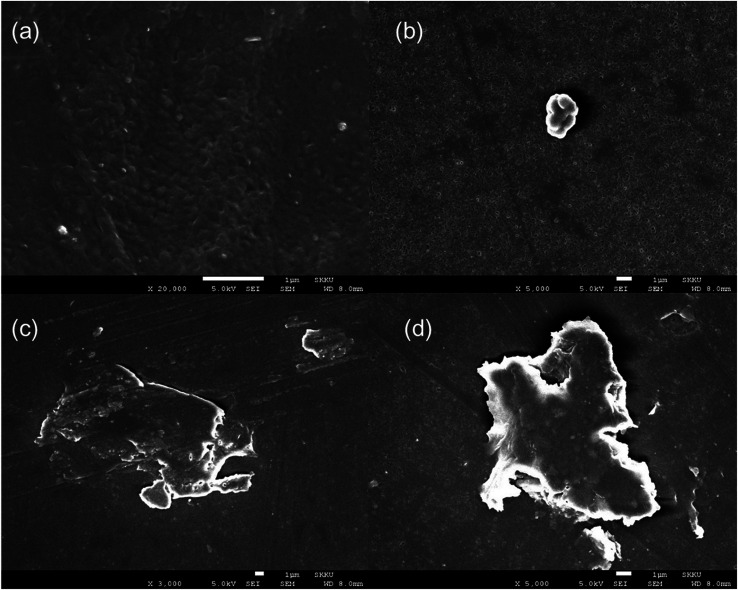
SEM images of surface of stereocomplex and stereocomplex/silane blend films: (a) stereocomplex; (b) MTMS 2.5 wt%; (c) ICPTES 2.5 wt%; (d) APTES 2.5 wt%.

The morphology of the silane-toughened PLA films was analyzed using SEM. The presence of PLA-grafted-silane was confirmed from SEM images as shown in Fig. 6. The surface of the PLA stereocomplex film is relatively smooth without any gel or rubbery particles (Fig. 6a). PLA/PLA-grafted-silane composite samples, however, exhibited very different topologies. Because there are no functional groups in MTMS, only silane-nano particles were present in the PLA matrix without any side linkage or dispersion (Fig. 6b).

**Fig. 7:**
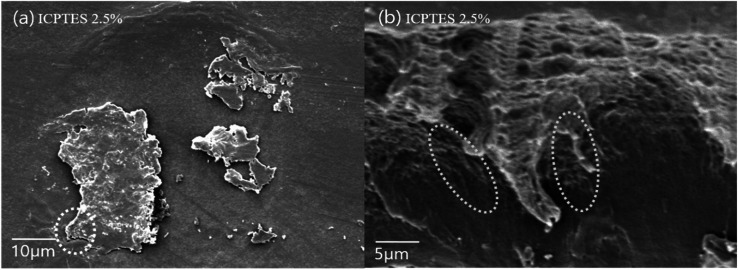
SEM images of the surface of (a) stereocomplex/ICPTES 2.5 wt% blend film; (b) magnification of the part shown in dotted line at (a).

The best dispersion of PLA-grafted silane rubbery gel was observed for the ICPTES/PLA composite, which contains characteristic cross-linked nanonetwork between the PLA matrix and PLA-grafted-polysiloxane, also referred as were silane rubbery gel, cross-linked network clearly was observed using cross-sectional SEM images (Fig. 7b). Micro and nano-sized-network in PLA-stereocomplex matrices improved the mechanical properties can be observed very clearly from the cross-sectional SEM image of ICPTES 2.5% samples. Those cross-linked structure and the distribution of silane rubbery gel at the nano-scale are mainly responsible for the relatively higher toughness. These results were also supported by the DMA and UTM analyses in this study. It was assumed that the isocyanate functional groups in the ICPTES reacted with end groups of stereocomplex PLA chains and formed a micro and nano-sized silane rubbery gel, which contained a core of silica and a brush-type shell of PLA. The brush or shell type fibrils and cross-linked network in the matrix, both are responsible for the increased in the ductility by forming interactions in the PLA matrix and increased the elasticity by decreasing the *T*_g_ of the polymer.^42^

Even distribution of a nanonetwork of ICPTES-rubbery gel was observed from SEM images of 2.5%, as shown in Fig. 7b. Also, micro-sized particles were observed in the stereocomplex films of APTES/PLA composites, as shown in the SEM image of the 2.5% APTES/PLA stereocomplex film in Fig. 6d. Furthermore, the reactivity of amine functional groups should be comparatively higher than that of MTMS but considerably lower than that of APTES. It was assumed that higher ratios of silanes have the tendency to form larger particles and aggregates, as it was clear from the mechanical analysis of 5% addition of silanes in the composites, where mechanical properties decreased noticeably compared to those at 2.5% addition.

**Fig. 8:**
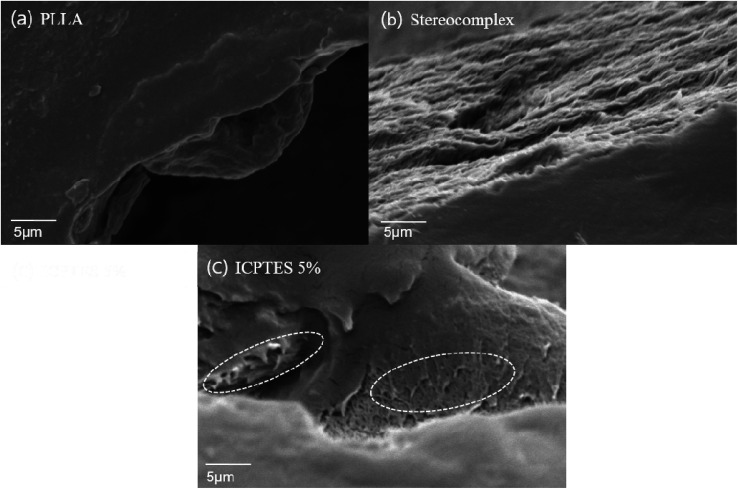
SEM images of the fractured surface of (a) PLLA; (b) stereocomplex; (c) stereocomplex/ICPTES 5 wt%.

Polymer toughening with addition of rubbery particles or plasticizers is frequently related to the formation of craze and micro or nano fibrils formation under applied stress during testing. The SEM images of fractured surfaces of neat PLA stereocomplex and PLA stereocomplex/PLA-graft-silane (ICPTES) blend samples are shown in Fig. 8. The fracture surface of neat PLLA showed a relatively smooth surface at the point of fracture (Fig. 8a). Similarly, the fractured surface of PLA stereocomplex showed slightly fibrils but brittle nature as both PLLA and PLA stereocomplex were fractured abruptly without any necking. However, stereocomplex exhibited a totally different array in the fracture area representing craze, cavitation and micro, and nano-fibrils. The micro and nano-sized cavitation and fibrils plays an important role during stress application on the material and contribute to dissipate a large amount of energy before fracture.^51^ This result in the increased toughness and improved tensile strength as shown in Fig. 8c. The nano-fibrils are formed due to the PLA polymeric chains grafting on the surface of silane particles which formed by condensation reaction. The results are in accordance with the previously reported results with similar silanes.^34^ Fibrillation support ductility by chain slippage and ease in chain motion under applied stress. The phenomenon has been observed in many polymer composites and blends and reported previously. The nanonetwork of nanofibrils which formed only in the silane blended PLA stereocomplex matrices enhanced the mechanical properties and elasticity as shown in a representative SEM image of ICPTES 5% films (Fig. 8c). Therefore, the nanonetwork of nanofibrils contribute to super-toughened of PLA. It is proposed that the functional groups on the ICPTES and APTES, isocyanate and amine respectively reacted with hydroxyl end groups of PLA and formed a rubbery core–shell structure within the highly crystalline matrices of PLA stereocomplex as it is clear from representative SEM images, see Fig. 7(a) and (b). The rubbery core was formed due to silane–silane linkages, whereas, surrounding core was formed by longer PLA chains by reacting hydroxyl ends with functional groups of silanes. However, in case of MTMS there is no functional groups at all, so no cross-linked network, see Fig. 6(b), only alkoxysilane is condense to form star a silane-based rubbery particle, similar particle types have been observed with different polymeric composite in already reported results.^52,53^ The addition of PLA-grafted silane in the PLA stereocomplex play important roles to enhance toughness such as silane form PLA-grafted-interconnections network within the PLA stereocomplex matrices as a result ductility increase due to chain slippage can occur under applied stress, the rubbery nature of silane condensate particles help to enhance the elasticity. It was further observed that higher contents of silane results to form lager particles due to its tendency to form aggregates, which resulted in deterioration of mechanical properties shown in Fig. 4, where elongation at break and yield strength was decreased considerably with addition of 5% silane in PLA stereocomplex.

From Fig. 8, it is confirmed that the difference morphology behavior of the tensile breaking area. In the fractured area, neat PLLA showed brittleness which related with tensile strength result of PLLA. However, stereocomplex and ICPTES 5% showed different characteristic compared to homo PLLA. Because of interconnection within stereocomplex chain, matrix of stereocomplex increased the elasticity compared to PLLA (Fig. 8b). From Fig. 8c, it is confirmed that the nanonetwork of nanofibrils were formed in the ICPTES 5% matrix. The nanonetwork of nanofibrils which formed only in the silane blended matrix enhanced of mechanical property and elasticity of ICPTES 5% films (Fig. 8c). Therefore, the nanonetwork of nanofibrils contribute to super-toughened of PLA.

Thus, we proposed an approach to modify the conventional PLA for high-end applications in biodegradable super-tough polymers with a broad scope of applications. This approach can improve toughness and other mechanical properties synergistically by cross-linked nano/micro-network formation in the stereocomplex matrix.

Many researchers have adopted synergistic approach using different compounds to enhance mechanical endurance.^54,55^ However, we have adopted a synergistic approach by targeting the intrinsically exhibited property of PLA, the stereocomplex formation combined with formation of *in situ* PLA-grafted-silane nano-gel to improve overall mechanical properties and toughness of PLA/silane composites. SEM images clearly show a rubbery gel within the PLA matrix by addition of ICPTES and APTES. The results also show that addition of MTMS only creates aggregates by condensation instead of formation of a PLA-grafted rubbery gel by *in situ* grafting. When comparing APTES and ICPTES, the miscibility and performance of ICPTES were very high. Interactions between silanes and the stereocomplex can form a cross-linked microstructure and enhance the mechanical properties of the composite.

## 4. Conclusion

Super tough PLA was obtained synergistically using the strength of a stereocomplexed PLA and the flexibility of a crosslinked network of organoalkoxysilane in the PLA matrix. Initially, PLLA and PDLA were synthesized by ring-opening polymerization. The molecular weights of the prepared polymers were characterized using GPC. A stereocomplex of PLA was prepared by solution casting and was characterized based on morphology and mechanical and thermal properties. Later, the stereocomplex was plasticized using *in situ* condensation and grafting of PLA with three different organoalkoxysilanes. Strain was improved significantly without significant loss of tensile strength or modulus. The results confirmed that PLA-*grafted*-ICPTES rubbery gel showed the highest toughness of the tested silanes. Moreover, up to 2.5% addition of ICPTES showed the most promising results with an elongation at break up to 100% without any significant loss in tensile strength or modulus, as confirmed from the mechanical results. The characteristic enhanced thermal stability and mechanical properties of stereocomplex PLA with improved toughness by PLA-*grafted* silane rubbery gel make this approach viable to produce very tough biodegradable materials for many applications.

## Conflicts of interest

There are no conflicts of interest to declare.

## Supplementary Material
